# Arenaviruses: Old viruses present new solutions for cancer therapy

**DOI:** 10.3389/fimmu.2023.1110522

**Published:** 2023-03-24

**Authors:** Paweł Stachura, Olivia Stencel, Zhe Lu, Arndt Borkhardt, Aleksandra A. Pandyra

**Affiliations:** ^1^ Department of Pediatric Oncology, Hematology and Clinical Immunology, Medical Faculty, Heinrich-Heine-University, Düsseldorf, Germany; ^2^ Department of Molecular Medicine II, Medical Faculty, Heinrich Heine University, Düsseldorf, Germany

**Keywords:** virotherapy, arenaviruses, LCMV (lymphocytic choriomeningitis virus), immunomodualtors, cold tumors, noncytopathic virus

## Abstract

Viral-based cancer therapies have tremendous potential, especially in the context of treating poorly infiltrated cold tumors. However, in tumors with intact anti-viral interferon (IFN) pathways, while some oncolytic viruses induce strong innate and adaptive immune responses, they are neutralized before exerting their therapeutic effect. Arenaviruses, particularly the lymphocytic choriomeningitis virus (LCMV) is a noncytopathic virus with preferential cancer tropism and evolutionary mechanisms to escape the immune system for longer and to block early clearance. These escape mechanisms include inhibition of the MAVS dependent IFN pathway and spike protein antigen masking. Regarding its potential for cancer treatment, LCMV is therefore able to elicit long-term responses within the tumor microenvironment (TME), boost anti-tumor immune responses and polarize poorly infiltrating tumors towards a hot phenotype. Other arenaviruses including the attenuated Junin virus vaccine also have anti-tumor effects. Furthermore, the LCMV and Pichinde arenaviruses are currently being used to create vector-based vaccines with attenuated but replicating virus. This review focuses on highlighting the potential of arenaviruses as anti-cancer therapies. This includes providing a molecular understanding of its tropism as well as highlighting past and present preclinical and clinical applications of noncytophatic arenavirus therapies and their potential in bridging the gap in the treatment of cancers weakly responsive or unresponsive to oncolytic viruses. In summary, arenaviruses represent promising new therapies to broaden the arsenal of anti-tumor therapies for generating an immunogenic tumor microenvironment

## Introduction

Recognition of the importance of the immune system in tumor surveillance has revolutionized the therapeutic landscape with the advent of immunotherapies such as checkpoint inhibitors (CI) (1). Despite some breakthroughs, tumor immune evasion provides obstacles to effective CI and/or other immunotherapeutic treatments focused on T cells and based on enhancing adaptive immune responses. These obstacles are commonly driven by an unfavourable tumor microenvironment (TME) milieu. Specifically, exhausted/dysfunctional T cells, an abundance of immunosuppressive tumor-associated macrophages (TAMs), monocytes and regulatory T cells (Treg’s), ineffective innate immune responses, poor immune cell infiltration and downregulated antigen presenting machinery within the TME contribute not only to CI unresponsiveness/resistance but generally immune evasion (2). Therefore, to elicit responsiveness to immunotherapies, the conversion of poorly inflamed cold tumors into hot tumors is therapeutically attractive and an area of active research (3). Strategies to induce this cold to hot conversion within the TME are numerous and can include innate immune activation (4), increasing MHC-I expression in tumor cells (3) and the use of viruses as anti-cancer agents (5).

The use of viruses as anti-cancer agents has been particularly relevant in recent decades as viruses are ideal vectors for gene therapy approaches and have been successfully applied in virus-based therapeutic vaccines as well as cell-based vaccines (6). Virus-based anti-tumor vaccines involve a combination of tumor-specific antigens, co-stimulatory proteins and immunomodulating molecules which boost the immune system to elicit anti-tumor responses (6). Examples in clinical development include the TG4001 modified vaccinia virus Ankara (MVA) vaccine encoding the HPV16 antigens and the interleukin 2 (IL-2) gene (7). Virus engineered cell based vaccines are centred on more personalized approaches and modify a patient’s immune cells *ex vivo* using viral vectors. Notable examples include the recently approved YESCARTA and KYMRIAH both of which target a patient’s T cells with a retrovirally inserted anti-CD19 Chimeric Antigen Receptor (CAR) for the treatment of non-Hodgkin lymphomas and acute lymphoblastic leukemia, respectively (8, 9). There are currently over three hundred clinical trials testing the efficacy of CAR-T cell therapy (6). The use of oncolytic viruses that preferentially replicate within the TME causing subsequent tumor cell lysis (10) and anti-tumoral activation of the adaptive immune system is another promising approach. Rigvir, the first approved oncolytic virus (in Latvia since 2014), is a genetically unmodified enteric cytopathic human orphan virus type 7 (ECHO-7) strain selected for melanoma (11). Another virus, Oncorine, is a modified adenovirus, approved in China for head and neck cancer (12) while Talimogene laherparepvec (T-Vec) is an HSV-1 based oncolytic virus that is currently in FDA approved for the treatment of recurrent melanoma (13). Over a hundred more are currently in the late and early stages of clinical testing. One challenge pertaining to oncolytic virus-based therapies is the induction of strong innate and adaptive anti-viral immune responses, especially the induction of type I interferons (IFN-I), which leads to clearing the virus before reaching its full therapeutic effect. In addition, patients previously vaccinated against and/or infected with related viruses have pre-existing T and B cell immunity including neutralizing antibodies which also results in fast virus clearance (14, 15). In stark contrast, the lymphocytic choriomeningitis virus (LCMV) is a non-oncolytic arenavirus currently in pre-clinical and clinical development, either as an anti-cancer agent or tumor vaccine vector, respectively. Infection with LCMV does not kill host cells by direct lysis and results in strong innate and adaptive immune responses also within the TME to eradicate the tumor. Compared to most oncolytic viruses, LCMV’s replication is not curbed by IFN-I (16), and its late induction of neutralizing antibodies allows for a more persistent intra-tumoral virus load to maximize effects on the TME (17). Taken together, viruses such as oncolytic viruses and certain arenaviruses represent a rich resource of potential novel anti-cancer therapeutics and this review aims to summarize the recent application of arenaviruses in cancer therapy and the potential gaps to be filled where other therapies are ineffective.

## The biology of arenaviruses

The *Arenaviridae* family consists of three genera, *Mammaarenavirus, Reptarenavirus* and *Hartmanivirus*, the first of which infects mammalian hosts. The *Mammaarenavirus* genus consists of 41 distinct viral species capable of infecting mammalian hosts and is geographically, genetically and epidemiologically sub-divided into Old and New World groups (18). Notable representatives of Old Word arenaviruses that will be mentioned in the current review include the LCMV strains, which were the first arenaviruses to be described in the 1930’s. Examples of New World arenaviruses which, in contrast can cause severe Haemorrhagic fevers include for example the Junin virus (JUNV) (causing Argentine Haemorrhagic Fever, AHF) and the Tacaribe virus (19).

**Figure 1:**
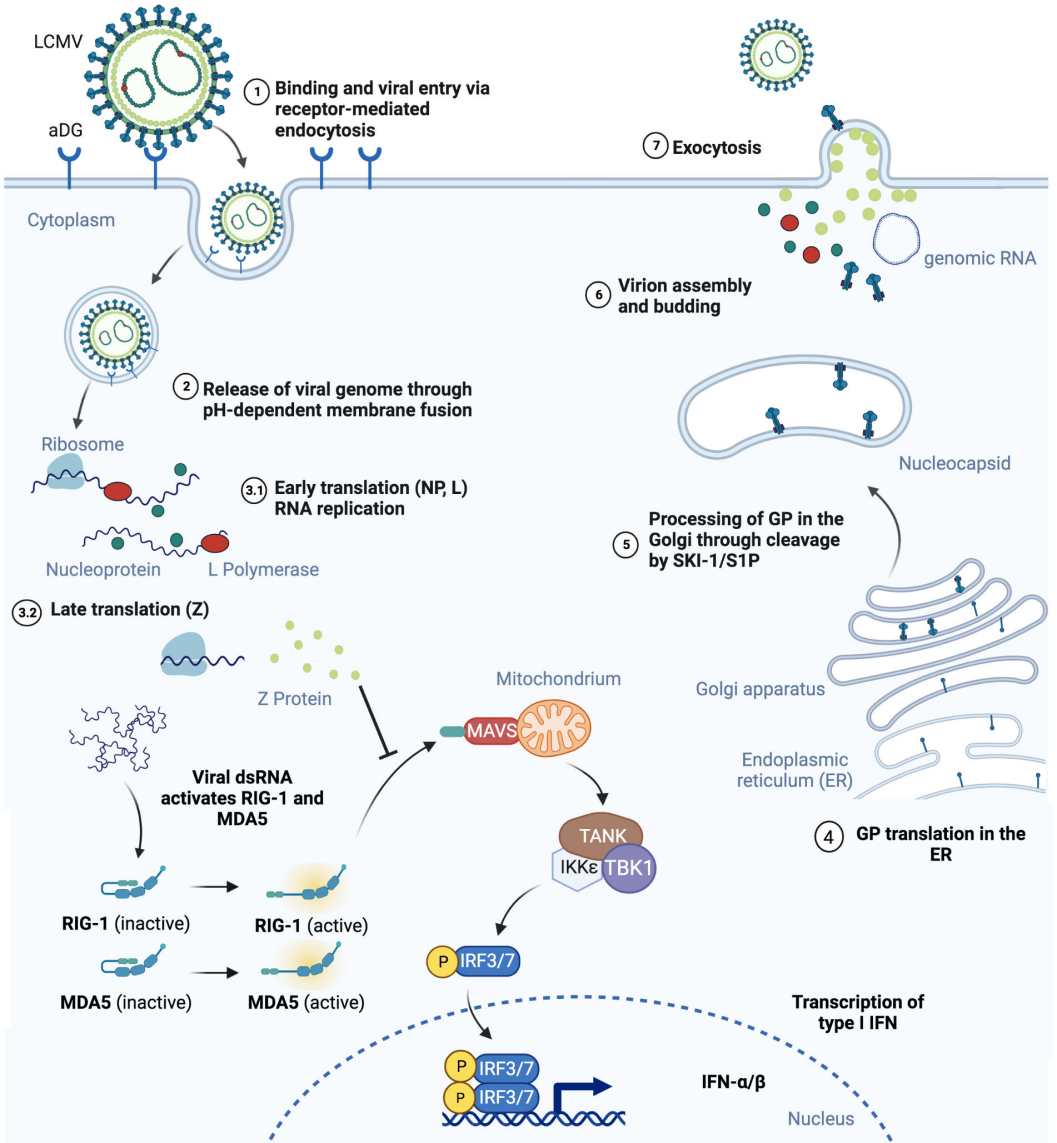
Schematic representation of mammalian cell infection by LCMV is shown. (1) LCMV endocytosis is αDG-mediated and (2) leads to the release of viral genome in the cell cytoplasm. (3) NP, L and Z proteins are produced. Virus RNA activates RIG-1 and MDA5, however binding to MAVS and its activation is blocked by Z protein, therefore inhibiting the INF pathway. (4) GP precursor translation takes place in the endoplasmic reticulum and (5) maturates in Golgi by SKI-1/S1P mediated cleavage. (6) NP, L and Z proteins together with viral RNA assembly into virions with GP on the surface and (7) bud out of the infected cell. Figure was created with BioRender.com.

The genome of arenaviruses is bi-segmented and composed of two single-stranded negative sense RNAs. The arenavirus lifecycle detailed in **Figure 1** is limited to the hosts’ cytoplasm and viral entry can be clathrin-dependent. Viral entry is mediated by the surface receptor α-dystroglycan (αDG) and CD164 for LCMV as well as Lassa virus (LASV) (20), and the human transferrin receptor 1 (Tfr1) for the JUNV and Tacaribe viruses (21). The wide spectrum of pathogenicity among the arenaviruses has been attributed to several factors. Arenaviruses use different receptors including αDG, human transferrin receptor 1, the transmembrane protein neuropilin 2 (NRP2) (22) and possibly additional proteins for viral entry. Differences in receptor distribution determine cell tropism. LCMV, for instance, which uses the ubiquitously expressed αDG for viral entry, can infect many cell types. However, it has been recently suggested that some arenaviruses including LCMV and LASV may use a combination of receptors or host factors including heparan sulfate proteoglycans or CD164 for viral entry (23–27). Differences in binding affinity of LCMV strains to αDG were previously proven to correlate with virus persistence and disease outcome. The Armstrong, E350 and WE2.2 strains with low affinity to αDG preferentially infect cells within the red pulp of the spleen and were not detectable in mice 7, 14 or 30 days post infection (28). In contrast, the Clone 13, Traub, and the WE54 strains with high affinity to αDG replicate in the white pulp of the spleen and are able to persist in mice, leading to chronic infection (28, 29). Bonhomme et al., through deletion of multiple GP1 and GP2 glycosylation sites that occur in different LCMV strains, were able to demonstrate that posttranslational modification differences of these proteins play an important role in virus fitness and ability to infect epithelial cells, macrophages or primary neurons (30). In addition to this differential use of binding receptors between arenaviruses, effects incurred by virus binding can also elicit additional changes. LCMV binding to αDG for example can lead to membrane destabilization and receptor downregulation, which can influence the future course of viral infection (23, 31). Furthermore, differences in cellular requirements enabling endosomal trafficking dependent or not on cholesterol, clathrin or caveolin (32–34) and immune evasion mechanisms also determine the pathogenicity during the course of arenaviral infection.

The innate and adaptive immune responses triggered by arenaviruses are critical for eventual viral clearance and these include IFN-I induction and the mounting of effective effector CD8^+^ T cell responses. Arenaviruses have developed several evolutionary mechanisms of evading immune detection. Binding to the retinoic acid-inducible gene I (RIG-I) and melanoma differentiation-associated gene 5 (MDA5) by the Z protein of New World arenaviruses prevents its association with mitochondrial antiviral signalling protein (MAVS) and blocks type I interferon beta (IFN-β) production (35). The NP protein in many arenaviruses including LCMV inhibits interferon regulatory factor 3 (IRF3) activation. Decreased IFN-β production has also been shown to occur through decreased PKR signalling (36–38). Eschli et al. demonstrated that the LCMV WE strain is only able to engage B cells with high viral loads due to a low frequency of GP1 specificity and sensitive epitope masking by glycosylation of the virus spike protein, which leads to weak antibody binding and, therefore, escape from early neutralisation (39). Taken together, considerable insights into the genetics, structure and life-cycle of arenaviruses has enabled their application into diverse research areas from investigating T cell dependent anti-viral immunity to their development as anti-tumor agents.

## Arenaviruses as anti-tumor agents

### Experimental, pre-clinical and clinical development of LCMV

For decades, LCMV has been the prototypic experimental arenavirus of choice for immunologists. Not only does infection with LCMV result in robust CD8^+^ effector T cell responses but also in long-term immunity. Indeed, its wide experimental use has led to monumental discoveries such as MHC restriction and PD-1’s role during T cell exhaustion (40). Checkpoint inhibition of the PD1-PD-L1 axis using monoclonal antibodies (mAb) such as the approved Nivolumab and Pembrolizumab has revolutionized the treatment landscape and ushered a new era of cancer immunotherapy (41, 42). Furthermore, by expressing LCMV-specific epitopes on tumor cells, it has been possible to study various aspects of CD8^+^ T cell mediated anti-tumor immunity (43, 44). In addition to being a useful biological tool, LCMV strains, through their immune-activating effects, have direct anti-tumoral effects (17).

**Figure 2:**
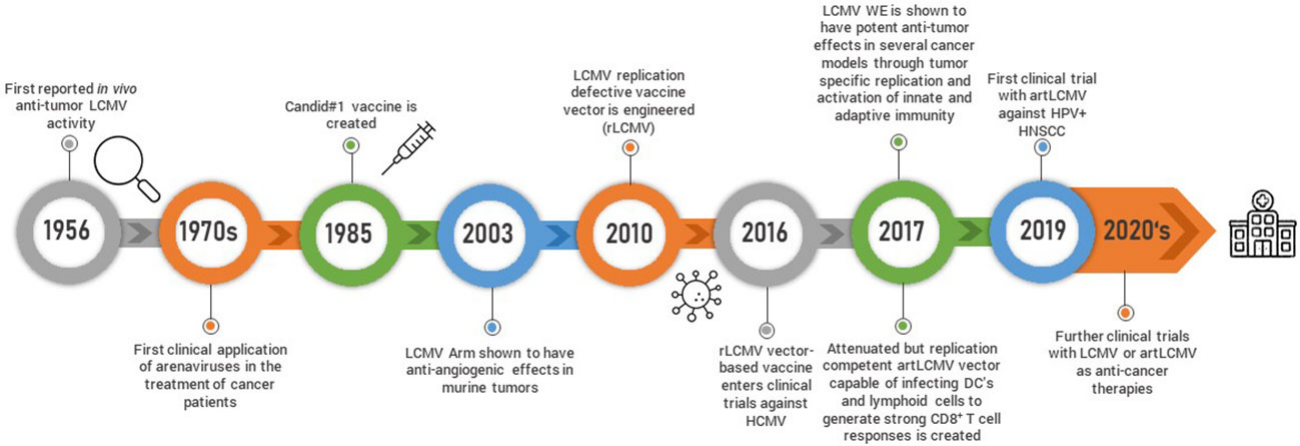
Schematic timeline representing arenavirus-based discoveries and research development is shown.

The observation that LCMV influences tumor growth dates back to sixty years ago when Nadel and Haas tested the efficacy of different strains of LCMV against the L1210 leukemia model in guinea pigs and mice (45) (**Figure 2**). Guinea pigs subcutaneously administered LCMV as late as seven days post tumor inoculation survived longer than their uninfected counterparts although this was not recapitulated in mice who succumbed to these particular LCMV strains. Fifteen years later, another group treated mice with LCMV and found that it potentiated the chemotherapeutic effects of 5-Fluoruracil (5-FU) (46). These observations with LCMV and similar studies with the MP virus (47), which is antigenically, morphologically and serologically considered to be a strain of LCMV (48), led to the treatment of cancer patients with the MP virus in the 1970s. Three patients with far-advanced lymphoma were intravenously treated with a single dose of the MP virus. All patients had underlying complications and were already pre-treated with several rounds of chemotherapy. One of the patients died from underlying pulmonary bacterial infections, another from pulmonary failure and a third one from disease progression (49). It is difficult to ascertain potential efficacy in such a small cohort of patients with very advanced disease. However, there was another larger clinical trial composed of 18 patients with more diverse though still advanced and pre-treated metastatic malignancies where the MP virus was administered *via* the intravenous route (50). None of the patients experienced any virus-induced encephalitis and three patients were not successfully infected. Out of the remaining 15 patients, 6 patients experienced a beneficial clinical response and/or presented evidence of tumor burden decrease. Meanwhile, with the advent of sophisticated genetic approaches and an increased understanding of the molecular, biological and immunological basis of viruses, the ability to better apply arenaviruses as anti-cancer agents has increased.

**Figure 3:**
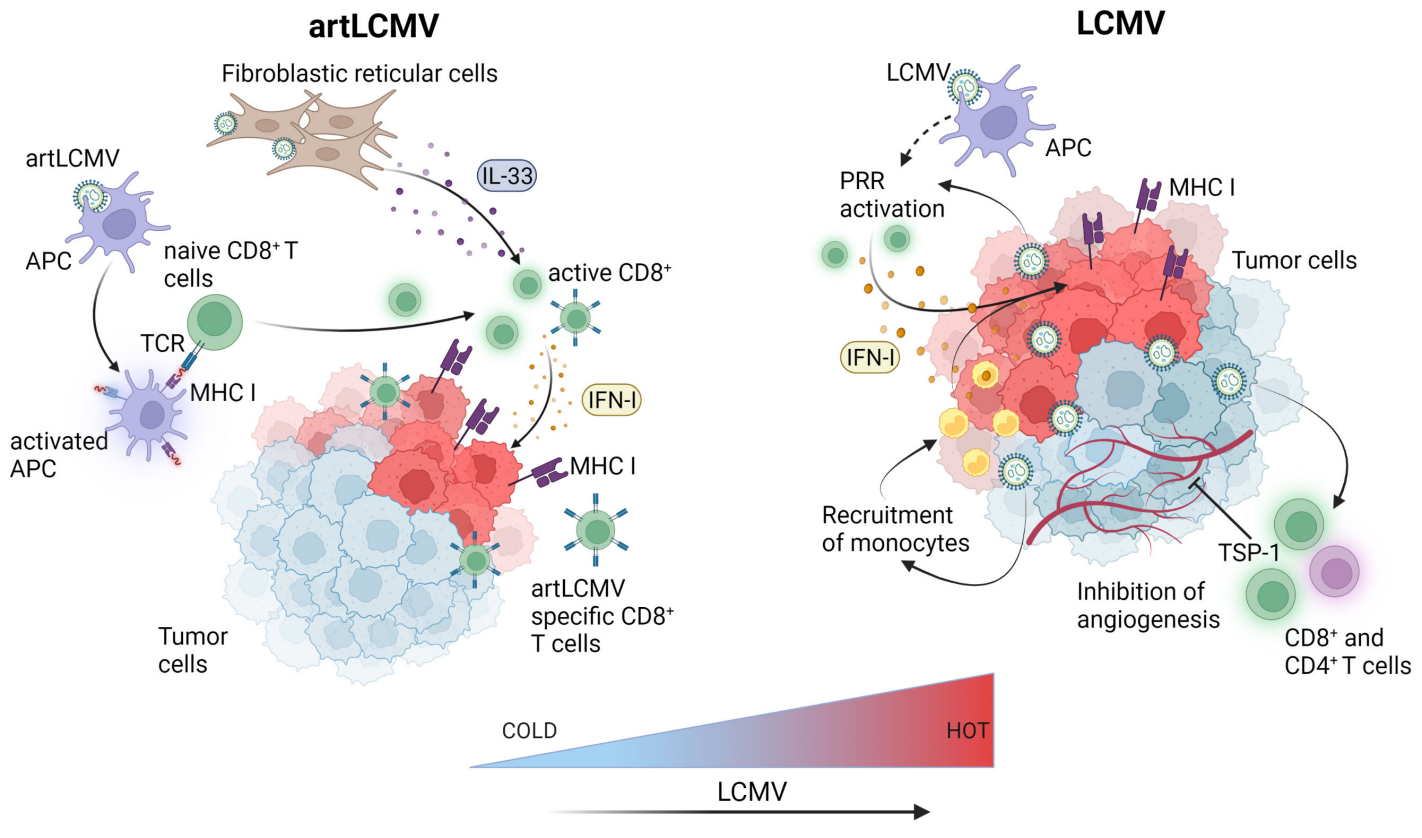
LCMV-based immunotherapies induce innate and adaptive immune responses within the tumor microenvironment. Attenuated but replicating artLCMV infects and activates APCs and delivers engineered tumor antigens for direct presentation to specific CD8^+^ T cells thereby inducing adaptive immune responses. Fibroblastic reticular cell infection by artLCMV leads to IL-33 secretion, activating the alarmin pathway in CD8^+^ T cells. LCMV directly replicates in tumor cells and APCs leading to pattern recognition receptors (PRR) activation and production of IFN-I in the TME. This leads to monocyte and cytotoxic CD8^+^ T cell recruitment, increased antigen presentation and MHC-I upregulation on tumor cells. LCMV also induces angiogenesis inhibiting TSP-1 surface expression on CD4^+^ and CD8^+^ T cells. Figure was created with BioRender.com.

Recently, it was shown that intravenous or peritumoral injection of the LCMV WE strain in several syngeneic or spontaneous murine and human xenograft models of cancer, including subcutaneous, endogenous hepatocellular carcinoma and spontaneous MT/*ret* melanoma led to regression or complete elimination of early-stage pre-established tumors (17). Kalkavan et al. also demonstrated that LCMV preferentially replicates in tumor cells and metastatic sites leading to robust immune infiltration with some accompanying replication in the liver. LCMV replication within the tumor persisted for at least thirty days post-tumor inoculation and tumor regression was dependent on IFN-I production by tumor-infiltrating monocytes. Importantly, IFN-I did not blunt LCMV replication within the tumor, allowing for sustained innate immune activation and clearance of LCMV from other organs. The preferential tumoral LCMV replication led to tumor regression through several proposed and interconnected enhanced innate and adaptive anti-tumor responses within the TME including local IFN-I production through the engagement of pattern recognition receptors, direct IFN-I anti-tumoral effects, reduced angiogenesis, recruitment of monocytes and cytotoxic CD8^+^ T cells to the TME and enhanced MHC I antigen presentation (17) (**Figure 3**). LCMV WE was also demonstrated to be superior to oncolytic viruses, a chimeric variant of vesicular stomatitis virus (VSV-GP) and a recombinant TK-depleted vaccinia virus (rVACV). Furthermore, LCMV WE was suggested in this and another study to have a strong anti-tumoral effect, especially when combined with checkpoint inhibition (51). As many of the cancer cell lines tested in *in vivo* tumor models by Kalkavan et al. are responsive to the anti-tumoral effects of IFN-I and express elevated levels of interferon receptors, preferential replication of LCMV within the tumor cannot be attributed to defects in interferon signalling but rather to expression differences in host factors crucial for viral replication between normal and cancer cells (52). This is an important point as oncolytic viruses are generally sensitive to IFN-I and their efficient replication is usually dependent on tumors harboring defects in interferon signalling (53). Therefore, patients whose tumors are characterized by intact IFN-I signalling are less likely to respond to oncolytic viral therapy leaving a gap that could be filled with LCMV.

Other studies utilized the acute LCMV Armstrong (LCMV Arm) strain to activate the immune system (54–56). For example, the infection of melanoma tumor bearing mice with LCMV Arm significantly slowed tumor growth and also decreased tumor angiogenesis. The anti-tumoral effects were shown to be dependent on LCMV-Arm-induced upregulation of angiogenesis inhibitor thrombospondin-1 (TSP-1) in CD4^+^ and CD8^+^ T cells (54). In another study, mice with advanced melanoma experienced restored tumor MHC-I expression following LCMV WE treatment leading to enhanced anti-tumor CD8^+^ T cell responses and tumor regression (57). The LCMV WE strain was also used to demonstrate the importance of NK cells and certain chemokines for an effective anti-tumoral response (58). Taken together, LCMV has been shown to efficiently re-direct the innate and adaptive immune system to target tumors. In murine tumor models, LCMV was demonstrated to be safe and effective through a variety of non-immunogenic and immunogenic tumor models. Current work is focused on increasing LCMV’s tumor tropism to translate its strong potential as anti-cancer agent into an effective tumor treatment. Targeted evolution is used to increase LCMV’s infectivity to tumor cells by retaining or decreasing its uptake into healthy cells and, therefore, healthy organs. This is achieved by a specific selection of tumor-prone virus mutations using the so-called Fast Evolution Platform. The overall aim is to maximize the inflammatory signals within the tumor tissue and thereby activate several anti-tumoral immune effector mechanisms. This approach is presently being developed by Abalos Therapeutics (59) (**Table 1**).

**Table 1:** Summary of current arenavirus-based clinical trials.

Virus	Targeted tumor	Current clinical stage	Study moderator
MVA	HPV16-positive	Phase II	Transgene
LCMV	Solid tumors	Pre-clinical	Abalos Therapeutics
rLCMV with rPICV	HPV16-positive HNSCC	Phase II	Hookipa Pharma
rLCMV with rPICV	Prostate	Phase I	Hookipa Pharma

### Genetically modified LCMV and other arenavirus vaccines

While the use of unmodified LCMV has been shown to be effective in controlling tumors, genetically modified LCMV used either as a vaccine or vector delivering tumor antigens is another promising approach. Flatz et al. engineered an LCMV based replication defective vaccine vector by successfully replacing the GP open reading frame portion of LCMV with vaccine antigens (rLCMV) (60). Insertion of up to 2.6 kilobases of foreign genetic material was possible and the vaccine was tested with several different cytotoxic T lymphocyte (CTL) epitopes including OVA (rLCMV-OVA) and GP33 (LCMV-GP33) to establish proof-of-principle efficacy and immunogenicity in several disease models. Robust and antigen specific CD8^+^ T cells responses occurred when mice were vaccinated with rLCMV-OVA, and this was mediated through direct targeting and activation of dendritic cells (DCs) which are critical antigen presenting cells necessary for CD8^+^ T cell activation. Mice inoculated with B16.F10 melanoma cells expressing the CD8^+^ T cell epitope GP33 and treated with rLCMV-GP33 eight days post-inoculation survived longer than mice treated with adenovirus 5 GP33 (rAd-GP33) or vaccinia virus GP33 (VACC-GP33) vaccines. Importantly, unlike other viral-based vaccines including the adenovirus 5 against which rLCMV was directly compared, rLCMV failed to induce vector antibody immunity in mice and non-human primates (61) enabling repeated boosters. The rLCMV vaccine vector is being translated into the clinic and its incorporation into a vaccine against Cytomegalovirus (HB-101 Vaxwave^®^) has reached Phase II clinical testing.

Although the rLCMV-GP33 vaccine was shown to increase survival in tumor-bearing mice, it was reasoned that the ability to replicate and deliver anti-tumor signals to cells other than just DCs might prove even more efficacious against tumors. This led to the development of a replication competent but attenuated LCMV vector (artLCMV) capable of infecting not only DCs but also lymphoid stromal cells (62) (**Figure 3**). Unlike rLCMV, artLCMV, through spread and infection to lymphoid stromal cells, induced the IL-33 alarmin pathway which has been shown to be critical for effective anti-viral and other immune responses (63). The combined effect of generating strong CD8^+^ T cell responses using a transplantable OVA-expressing tumor model, IL-33 alarmin signalling and IFN-I production (for 48 hrs) led to more potent and specific anti-tumor immunity and subsequent tumor control superior to that of the replication deficient rLCMV without neutralizing antibody production (62). However, responses were still hampered by self-tolerance and strong responses against vectorized non-self antigens at the expense of tumor specific ones. To overcome this competition between tumor and vector specific cytotoxic effector T lymphocytes (CTLs) Bonilla et al. designed a 2-vector therapy system based on two distantly related arenaviruses (LCMV and Pichinde virus (PICV)). This strategy was able to reshuffle immunodominance in favor of tumor specific CTLs, which led to more effective tumor control and protection against tumor rechallenge (64). Attenuated replicating vector arenaviruses (TheraT^®^ platform) are in the clinical stages of commercial development for the treatment of prostate cancer (HB-301 TheraT^®^), HPV+ Head/Neck Cancer (single LCMV based HB-201 or in combination with PICV based HB-202 TheraT^®^) (**Table 1**). Much like the acute LCMV strains, the artLCMV platform stimulates innate immune responses and is also dependent on replication in antigen-presenting (APCs) cells to elicit its anti-tumor effects. The Phase I/II clinical trial (NCT NCT04180215) is an open-label study commenced in 2019 evaluating HB-201 and HB-201 and HB-202 as single or two-vector therapy in previously treated patients with advanced or metastatic HPV 16+ cancers, mainly head and neck. Recently, preliminary data from the trial reported the presence of E6/E7 specific CD8^+^ T cell levels in the blood and a high infiltration of CD8^+^ T cells in over 50% of patient tumor samples (65). One main disadvantage of this approach, however, is that this arenavirus platform currently only delivers the HPV16 epitope in the case of head and neck cancer, or targets the most common prostate cancer markers PAP, PSA, PSMA but cannot be used for other types of tumours unless novel antigens are specifically integrated.

### Safety and dosing of LCMV and arenavirus-based vector vaccines

Although the numbers of LCMV infected people are not known as only the most severe infected cases are reported, serological studies indicate that around 5% of the American (66), 1.7% of the Spanish, 2.9% of the Dutch and 0.3% of the French populations (67) have LCMV specific antibodies, indicating previous exposure to the virus. While some arenaviruses cause fatal hemorrhagic fevers, symptoms caused by LCMV infections are comparatively mild and include influenza-like symptoms as well as dysesthesia (23). This initial phase of disease symptoms when occurring may be followed by a symptom-free period of a few days up to 3 weeks, before the beginning of a second phase. The latter is characterized by fever, headache, nausea and meningeal irritation and is usually followed by complete recovery. This has been corroborated by well-documented cases of infected laboratory workers (68–70). Although LCMV does not pose a serious health risk in the general population, infection during organ transplantation and pregnancy can be detrimental. In one published case, three organ recipients receiving kidneys and liver of a donor developed virus infection symptoms including fever and encephalopathy soon after transplantation leading to death within 36 days. Analysis of the viral protein sequences revealed 14 fragments consistent with arenaviruses most closely related to LCMV (71). In another case, all organ recipients developed illnesses symptomatic of virus infection and a liver recipient died 2-3 weeks post donation. LCMV was later found in the aortic tissue of the donor and the infection was confirmed in the recipients thirty-seven days after transplantation (72, 73). The source for the donor infection was later identified to be pet animals such as a hamster, corroborating reports that direct human to human transmission does not occur (74). All of these severe effects of an unrecognized LCMV infection may be attributed to the concurrent treatment of the transplantation patients with immune suppressive drugs thereby not enabling an efficient anti-viral immune response at the time of infection. Detrimental effects of LCMV can also be observed during congenital infection which can severely affect the survival and well-being of the children affected. The most common symptoms of congenital LCMV infection are chorioretinitis, hydrocephalus and ventriculomegaly (75).

Since in clinical LCMV applications, an intravenous (IV) route of administration is preferred, off-target replication in organs other than the intended tumor or lymphoid organs (in the case of LCMV-based vector vaccines) should be considered. Preclinically, subcutaneous injection of LCMV WE resulted in detectable virus in the skin and spleen 8 days post infection in mice (17). Even after intravenous infection of mice, although dose dependent increases of liver enzymes were measured, changes were in all cases transient and enzyme levels returned to background levels ten to fifteen days after infection (76). In the context of replication competent arenavirus vaccine vectors, detection of the vector was apparent in the spleen and liver of mice but was rapidly cleared without induction of organ damage (62). However, in certain mouse strains including the virus-sensitive FVB or NZ, infection with LCMV Clone13 but not with other variants, like Arm, does result in severe illness including thrombocytopenia and hepatocellular necrosis (77). Such severe disease symptoms can be avoided not only by choosing the right LCMV strain, but also by virus attenuation for example by reassorting the genome segments of two different LCMV strains (77). Therefore, virus strains for clinical development will have to be carefully chosen to avoid any potential for more severe disease effects and carefully evaluated in respective animal studies. Taken together, preclinical *in vivo* studies suggest that for certain wildtype or recombinant LCMV strains while replication in off-target organs such as the liver and spleen occurs, the virus is rapidly cleared and does not persist long enough to induce adverse organ damage.

Clinically, the safety of administering therapeutic LCMV to potentially immune-suppressed and conceivably heavily pre-treated cancer patients needs to be carefully evaluated (49, 78). Previously, the administration of the MP LCMV strain to immunosuppressed cohorts with metastatic disease in the two clinical trials performed in the 1970s was generally well-tolerated and did not result in serious viraemia related side-effects (50). However, in the 1971 study after a single intravenous injection of the three advanced lymphoma patients, viral titers were detectible post-mortem in multiple organs in all the patients (49). Nevertheless, few virus-related adverse effects on normal tissues were observed pointing to a potentially favourable safety profile (50) which may even be further enhanced by the identification of tumor-tropic replication-competent strains. The above mentioned Phase I/II study is slated for completion in 2025, but initial reports of safety, tolerability, and immunogenicity are encouraging although so far 2 patients experienced dose-limiting toxicity involving Grade 4 hepatitis or Grade 4 encephalopathy (79, 80). A recent update presented at ASCO 2022, revealed plans to investigate a combination of HB-201 with pembrolizumab (81) in the Phase II portion of the trial.

Preclinically, LCMV and arenavirus vectors are able to elicit immune responses through several routes of administration including intravenous, intradermal and subcutaneous (ranging from 10^2^-10^6^ PFU per animal), with one dose often being sufficient to elicit effective anti-tumor immune responses in mice, albeit when evaluating tumor rechallenge and booster regimens, more doses may be required. Clinically, in the case of arenavirus vectors, both intravenous and intratumoral routes of administration have been applied, although the IV route enables secondary lymphoid organs to be reached. As LCMV and vector-LCMV neutralizing antibody production currently appears not a hindrance, repeated dosing where clinically necessary should be possible, although the potential for neutralizing anti-viral immune responses will have to be carefully explored during ongoing and coming clinical evaluation of LCMV cancer therapy.

### Live-attenuated Junin vaccine (Candid#1) and other arenaviruses

Before the development of the Candid#1 vaccine, infection with the hemorrhagic fever (HR) causing Junin virus resulted in the highest levels of mortality (15-30%) of any other HR causing arenavirus (23). The Candid#1 is a live attenuated vaccine and was generated through serial passaging of the Junin virus in guinea pigs followed by suckling mice and finally in tissue culture. Although its commercial distribution is limited due to the relatively small affected Argentinian population, it has been an effective vaccine in protecting against Junin virus infection. Recent hints into the molecular mechanism of Candid#1 attenuation point to a single residue change F427I in the G2 transmembrane domain of the GP leading to decreased virulency (82). At the same time, this may limit its more wide-spread use as a vaccine due to the potential of back mutation, and therefore, other approaches to develop vaccines targeting arenaviruses inducing hemorrhagic fevers are currently exploited including the addition of more attenuating mutations (e.g., for Junin) (83) or genome reassorting from hemorrhagic and non-hemorrhagic arenavirus strains (e.g., Lassa and Mopeia) (84, 85).

Kalkavan et al., in addition to uncovering the already mentioned anti-tumoral effects of LCMV strain WE, found that, following injection, the Candid#1 vaccine also replicated within tumors and decreased xenograft tumor growth of human cancer cell lines in NOD.SCID mice (17). However, the *in vivo* anti-tumoral effects of Candid#1 occurred following direct intratumoral injections and it is currently unclear whether the attenuated virus would preferentially replicate in the tumor if applied by a more clinically relevant application route. An *in vitro* study found that Candid#1 was cytopathic and induced apoptosis in several human cancer cell lines in an interferon independent manner, linking the mechanism to RIG-I with higher viral replication in RIG-I deficient cell lines or after knocking it down (86). Apoptotic effects on normal cell lines however, were not tested and the study was limited in the number of cell lines used. Despite the preliminary nature of the above studies, they are nevertheless promising. The Candid#1 vaccine has already been successfully and safely used in humans, and next generation approaches are currently underway. Although approval from the Food and Drug Administration (FDA) in the US is still pending, it has been produced and used on a larger scale by the Argentinian government. As the attenuated phenotype of the Candid#1 vaccine appears to be based on the single substitution at residue 427 (F427I), the FDA’s primary concern with the vaccine has been a potential reversion to its previously virulent phenotype. Indeed, serial passaging of the Candid#1 virus in cell culture can lead to reversion (87) and approaches in generating second-generation Candid#1 vaccines are focusing on inserting additional mutations into the virus’ GPC in order to create a barrier to reversion (83, 87). It is also worth mentioning that the Tacaribe virus, which is another New World arenavirus closely related to the Junin virus, was found not to be virulent (88). Wolf et al. discovered that infection of cancer cell lines and primary macrophages with the Tacaribe virus causes caspase-dependent apoptosis (89). Although the apoptosis was shown to depend on active viral replication, it was not further mechanistically investigated. It would be interesting to extend this finding in an *in vivo* setting and explore whether the Tacaribe virus would preferentially replicate within tumors and also have anti-tumor effects.

Another interesting approach was presented in a study by Muik et al. that used an oncolytic VSV virus with an exchanged surface glycoprotein of LCMV origin (VSV-GP) as an anti-tumor agent. Oncolytic viruses are usually rapidly neutralised, whereas VSV-GP appears to avoid neutralizing humoral responses by failing to induce nAb against the LCMV spike protein (90). Other efforts focused on exchanging the VSV glycoprotein with another New World arenavirus, Lassa (VSV-Lassa-GPC). VSV-GP and VSV-Lassa-GPC have shown pre-clinical efficacy in tumor models (91), and VSV-GP is currently evaluated in a Phase I study alone or in combination with checkpoint therapy (92).

## Concluding remarks and future perspectives

Arenaviruses, particularly the well-studied LCMV viruses, have a strong potential to make an impact in cancer therapy. The efficacy of LCMV, whether unmodified strains, recombinant strains with increased tumor cell tropism, or incorporated into a viral attenuated vaccine, in controlling tumors in a broad range of pre-clinical murine models of cancer has been demonstrated. As already shown by the multifaceted use of oncolytic viruses in cancer therapy, there appears a substantial potential for live replicating arenaviruses in the treatment of tumors. Unlike oncolytic viruses, LCMV preferentially replicates in a wide range of tumors and can robustly continue to do so even in tumors where interferon signalling is intact. Furthermore, induction of IFN-I by LCMV does not curb viral replication within the TME allowing for sustained immune activation and enabling control of the virus in normal tissues, thereby minimizing potential collateral damage and increasing the therapeutic index. The anti-viral immune responses elicited by LCMV in murine tumor models were shown to be instrumental in contributing to tumor regression and did not blunt the anti-tumor efficacy of the virus, which is another common challenge faced by oncolytic viruses. The production of neutralizing antibodies can suppress oncolytic virus efficacy but LCMV fails to elicit strong neutralizing antibody responses (39, 93). Instrumental to translating LCMV to the clinic will be a thorough safety evaluation, and a deeper understanding of the underlying mechanisms of tumor replication and anti-tumoral effects. Studies of viral entry receptor distribution in tumor cells and a closer examination of the specific host factors in tumor cells which enable LCMV replication might lead to further mechanistic insights and shed light on how to optimize LCMV treatments and uncover responders to therapy. The fact that LCMV variants were already administered to human patients decades ago resulting in responses in some patients is encouraging in paving the pathway to future applications of the virus as an anti-cancer therapeutic.

The artLCMV vaccine platform is one of the anti-cancer therapeutic approaches in pre-clinical and clinical development. The low seroprevalence of LCMV in the general population (67), coupled with weak neutralizing antibody production against LCMV (93) appears to allow for repeated application which is yet a substantial limitation of many other viral based vaccines and oncolytic viruses, and might enable higher patient response rates. The artLCMV’s anti-tumoral mechanism of action depends on the infection of APCs to elicit CD8^+^ T cell responses and to activate the IL33-alarmin pathway in lymphoid tissue. It remains to be further investigated whether effective anti-tumoral cytotoxic effector T lymphocyte (CTL) responses can be successfully recapitulated in a clinical setting in those tumors where the expression of tumor specific-antigens or neoantigens may be a limiting factor in the successful induction of CTL responses due to tumor heterogeneity, evasion mechanisms including loss of target antigen, downregulation of MHC molecules and T cell exhaustion (94).

On the other hand, LCMV as a cancer therapy currently developed in the absence of a vaccine antigen has the advantage that the concurrent administration of tumor-specific or neo-antigens is not required for an efficient anti-tumoral response. Although LCMV is not cytopathic, it induces strong innate and adaptive anti-tumoral responses including the local activation of pattern recognition receptors within the TME, which in turn allow for IFN-I and inflammatory cytokine production, immune cell infiltrating recruitment and the release of tumor neoantigens and subsequent generation of adaptive immune responses. The induction of innate immune responses can counteract immunosuppressive tumor-promoting mechanisms within the TME. This makes such LCMV strains more broadly applicable than the artLCMV platform and not contingent on treating tumors with well-defined stably expressing tumor antigens. Although still in their infancy, the use of other attenuated arenaviruses including live vaccines such as Candid#1 for the treatment of tumors might also hold promise especially if safer second-generation vaccines can be developed.

Finally, as with other virus based vaccines and oncolytic viruses, combinatorial approaches with other immunotherapies or anti-cancer agents will likely prove therapeutically effective, especially in treating poorly immune infiltrating cold tumors. The tumor TME of cold tumors is often characterized by high PD-L1 expression, low immune infiltrates including cytotoxic T cells and/or low expression of the antigen presentation machinery (95). That, when combined with low neoantigen levels makes these tumors largely unresponsive to immunotherapies. By contrast, tumors that are immunologically scored as “hot” are highly infiltrated with cytotoxic T cells and are more responsive to immunotherapies (3). Therefore, approaches such as arenavirus therapies that can successfully manipulate the TME towards an increased ‘hot’ phenotype (**Figure 3**) could not only lead to increased immunotherapeutic responses but open up previously poor candidate patient cohorts to immunotherapy treatment.

The first clinical trial combining an oncolytic virus therapy (T-Vec) with the anti PD-1 Pembrolizumab demonstrated that T-Vec promoted tumoral T cell infiltration improving Pembrolizumab’s efficacy (96). Patients with advanced melanoma in a phase II randomized study receiving a combination of T-Vec with the anti-CTLA-4 antibody ipilimumab experienced significantly higher objective responses than patients receiving ipilimumab alone (97). Unfortunately, combination therapy failed phase III, as there was no significant improvement in the survival of patients treated with addition of T-Vec (98). Virus based vaccines such as viagenpumatucel-L (gp96-Ig-secreting allogeneic tumor-cell vaccine HS110) in combination with the anti-PD-1 Nivolumab in patients with non-small cell lung adenocarcinoma successfully completed a phase II clinical trial (61). However, one of the major obstacles missing from the arsenal of current immunotherapy combinations, especially for vaccines, is the ability to selectively and specifically activate tumor-killing immune infiltrates for long enough to overcome the metabolic, spatiotemporal and immune barriers imposed by the immunosuppressive cells within the TME such as M2 macrophages, myeloid derived suppressor cells (MDSC) and regulatory T cells (Treg’s) which can cause anergy, exhaustion and senescence of cytotoxic lymphocytes as well as the induction of pro-tumoral inflammation. There exists a niche for LCMV-based arenavirus therapies, especially in the treatment of poorly infiltrating cold tumors as well as tumors with intact interferon responses, both instances where viral vaccines and oncolytic viruses, respectively, might have limited efficacy. Finally, the potential use of LCMV-based arenavirus therapies could boost the response rates of immunotherapies such as CI’s that rely not only on adequate CD8^+^ T cell infiltration but de-repression of immunosuppressive mechanisms within the TME.

## Author contributions

PS wrote sections of the manuscript, visualization, and editing. OS and ZL contributed editing and figures. AB proofread the manuscript and provided suggestions. AAP wrote, edited and supervised the study. All authors contributed to the article and approved the submitted version.
